# Glutamine metabolism remodels tumor-associated macrophage: mechanistic explorations and new strategies in translational medicine

**DOI:** 10.3389/fimmu.2025.1715170

**Published:** 2026-01-15

**Authors:** Juya Cui, Xia Yan, Jianbo Song

**Affiliations:** 1 Shanxi Bethune Hospital, Shanxi Academy of Medical Sciences, Third Hospital of Shanxi Medical University, Tongji Shanxi Hospital, Taiyuan, China; 2 Third Hospital of Shanxi Medical University, Shanxi Bethune Hospital, Shanxi Academy of Medical Sciences, Tongji Shanxi Hospital, Taiyuan, China; 3 ’ Shanxi Provincial Peoples Hospital Affiliated to Shanxi Medical University, Taiyuan, China

**Keywords:** glutamine metabolism inhibitors, glutamine metabolism, immune response, tumor microenvironment, tumor-associated macrophage

## Abstract

Glutamine metabolism provides energy and raw materials for tumor survival and proliferation. In addition to affecting cancer cells, many studies have investigated the role of glutamine metabolism on the tumor microenvironment in depth. The macrophages, which show high frequently distribution in the majority of solid tumors, are important immune cells in the tumor microenvironment. Tumor-associated macrophage metabolic network remodeling is enormous and complicated. This review links TAM with glutamine metabolism, and combs the network relationship between the unique functional state of macrophages and the key programs of glutamine metabolism in terms of epigenetic modification, signaling pathway transduction, and metabolic checkpoint regulation. In addition, we will summarize the existing glutamine metabolism-targeting drugs and explore the new technologies and strategies for glutamine metabolism to regulate the functional state of TAM.

## 1 Introduction

The tumor microenvironment (TME) is the complex environment surrounding tumor cells which regulates crucial aspects of tumor biology, including disease progression, metastasis, immune evasion, and treatment resistance (1).The core of the tumor microenvironment (TME) dynamics lies in its immunosuppressive milieu, typically driven by metabolic reprogramming of resident cells (2, 3). Metabolic reprogramming is a hallmark of cancer, a process that supports rapid growth and proliferation by ensuring an adequate supply of proteins, nucleotides, and lipids (4). Specifically, competition between cancer cells and immune cells for nutrients and metabolites such as glucose, lipids, glutamine, and lactate can lead to nutrient deprivation and metabolic reprogramming of immune cells, thereby regulating their function within the TME.

Amino acid metabolism is a crucial factor in the complex metabolic dynamics within TME. Glutamine, a non-essential or conditionally essential amino acid, plays a key role in clinical diseases and stress states. In cancer cells, glutamine not only provides a nitrogen source for amino acid and nucleotide biosynthesis but also serves as a carbon source to replenish the tricarboxylic acid (TCA) cycle and lipid biosynthesis pathways. Hence, cancer cells are addicted to glutamine (5). Some studies have shown that glutamine-derived αlutamine-deriv remodels M2 macrophages by increasing fatty acid oxidation and Jmjd3-dependent epigenetic reprogramming of M2-related genes (6). Glutamine metabolism plays an important role in macrophage polarization. And there are inherent differences in the dependence on glutamine metabolism among kinds of macrophage subsets.

Macrophages are important immune cells and play a significant role in both innate and adaptive immunity (7).As a key immune cell subset, tumor-associated macrophage (TAM) infiltrates TME, characterized by phenotypic plasticity and heterogeneity. This heterogeneity reflects the responsiveness of TAM to environmental stimuli, leading to polarization from pro-inflammatory (M1-like) to anti-inflammatory (M2-like) phenotypes. Specific subgroups of TAM are associated with various biological processes, such as cancer progression, angiogenesis, vascularization, immunosuppression, metastasis, treatment resistance, and poor clinical outcomes (8). Particularly, TAM plays a crucial role for the immunosuppressive nature of TME. The existing immune checkpoint inhibitors have limited efficacy against “cold tumors”, and the strategy targeting metabolic-immune crosstalk is feasible. Metabolic reprogramming is used to regulate macrophage polarization. It is necessary to elucidate the mechanisms behind metabolic processes and macrophage polarization, as well as to explore potential strategies targeting TAM.

Currently, the combined treatment with GLS1 inhibitor CB-839 has entered the clinical trial stage and achieved certain therapeutic effects (9). Although there is a growing awareness of the importance of metabolic crosstalk between cancer cells and TAM, the subtypes of TAM involved in metabolic remodeling to control tumor progression and their underlying mechanisms remain unclear and require more research. In addition, little is known about the molecular mechanisms linking glutamine metabolism to immune checkpoints such as PD-L1 and CD47.Consequently, delving into the reprogramming of glutamine metabolism in TAM and its implications for immune function is of great significance. This exploration can facilitate a deeper understanding of the mechanisms by which glutamine metabolism modulates TME and the body’s immune response to tumors. It is expected to uncover novel therapeutic targets and strategies, thereby offering new directions for the combinatorial treatments.

## 2 The characterization of glutamine metabolism and macrophage polarization

### 2.1 The core pathways and regulatory nodes of glutamine metabolism

Glutamine traverses the cell membrane via specific transporters (including SLC1A5, SLC38A1/A2, and SLC6A14) to enter the cells. Subsequently, with the assistance of SLC1A5, glutamine gains entry into the mitochondria. Once inside the mitochondria, glutaminase (GLS/GLS1) catalyzes the conversion of glutamine into glutamate. This conversion process represents the rate-limiting step in the catabolic degradation of glutamine. Glutamate is deaminated by glutamate dehydrogenase (GLUD),leading to the formation of α-KG (α-ketoglutarate). Additionally, in the presence of different enzymes, glutamate is metabolically diverted into other products. For example, under the action of glutamate oxaloacetate transaminase (GOT), glutamate is converted into aspartate. And under phosphoserine aminotransferase 1 (PSAT1), it is converted into phosphoserine, all while also generating α-KG (5).Finally, α-KG enters the tricarboxylic acid (TCA) cycle, during which the production of NADPH occurs (10).

**Figure 1:**
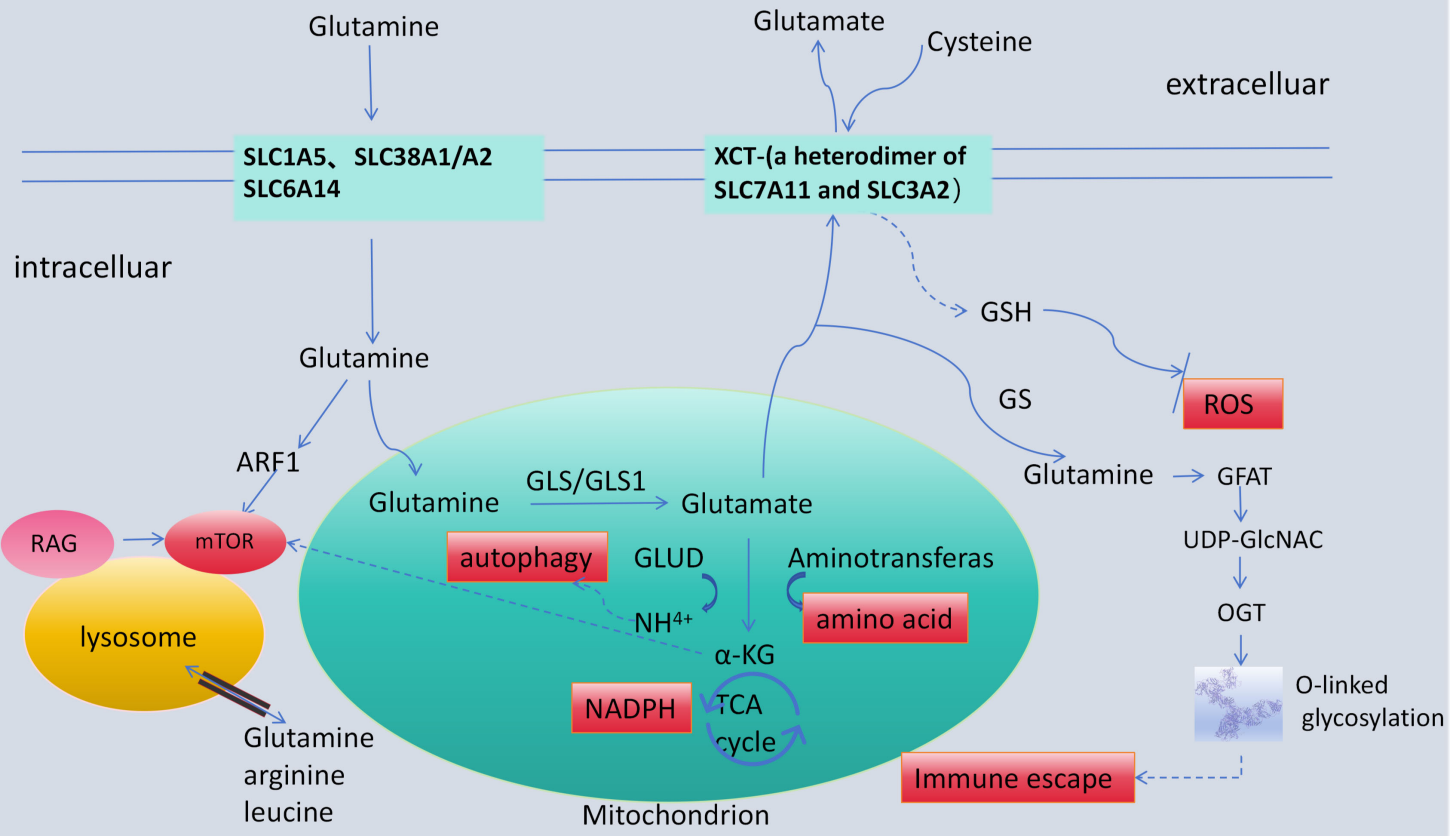
Schematic diagram and functional demonstration of glutamine metabolism in tumor cells. Rapid proliferation of tumor cells generates high levels of endogenous reactive oxygen species (ROS). Glutamine metabolism regulates ROS by promoting the synthesis of glutathione (GSH) and reduced nicotinamide adenine dinucleotide phosphate (NADPH). Cysteine which are transported into cells via the transporter XCT- (an heterodimer of SLC7A11 and SLC3A2), along with glycine and glutamate, forms GSH to neutralize ROS. Glutamine activates the mTOR pathway through ADP-ribosyltransferase 1 (ARF1), which regulates protein and fatty acid biosynthesis by sensing amino acids (including glutamine) while inhibiting autophagy-related degradation processes (15). The relationship between autophagy and glutamine metabolism is complex. Current consensus suggests that autophagy plays a dual role in tumor development. Glutamine inhibits general control nonderepressible 2(GCN2)activation and integrates stress responses (ISR), thereby promoting autophagy. It also indirectly stimulates mTOR to suppress autophagy by complex mechanisms. Additionally, cancer cells can induce autophagy using ammonia (5). Glutamine metabolism serves as a critical metabolic pathway during macrophage polarization. Glutamine deficiency inhibits M2 macrophages polarization by suppressing the biosynthesis of uridine diphosphate N-acetylglucosamine (UDP-GlcNAc) and the N-glycosylation of M2-related proteins such as Relmα, CD206, and CD301, while maintaining M1 polarization unaffected, thereby affecting immune evasion (6). glutaminase (GLS), glutamate dehydrogenase (GLUD), activating transcription factor 4 (ATF4), glutamine fructose-6-phosphate aminotransferase (GFAT), α-ketoglutarate (α-KG), tricarboxylic acid (TCA), and O-linked β-N-acetylglucosamine transferase (OGT).

In tumors, glutamine derived α-KG serves as a replenishing substrate to re - activate the inhibited TCA cycle in tumor cells, thus allowing cancer cells to rely on glutamine. This is termed the “recovery effect” (11, 12). α-KG generally engages in the TCA cycle via two mechanisms, mainly contingent upon oxygen supply levels and mitochondrial function (13). Under aerobic conditions, tumor cells with normal mitochondrial function can utilize α-KG for the complete TCA cycle, restarting the suppressed oxidative phosphorylation process to provide energy and raw materials for cellular growth and proliferation. During hypoxia or mitochondrial dysfunction, IDH1 or IDH2 - catalyzed reductive carboxylation reactions generate α-KG, which is then converted into citrate. Citrate can be exported from mitochondria to support lipid synthesis (14).Glutamine metabolism is reprogrammed in tumors. It not only supplies energy and carbon/nitrogen sources essential for biosynthesis but also participates in diverse biological processes, including maintaining redox homeostasis, signal transduction, epigenetic modification, autophagy regulation, and immune evasion, playing a crucial role in tumorigenesis and progression. (**Figure 1**).

Current researches on glutamine metabolism in tumors have provided a general understanding, particularly in terms of “how” and “what” to utilize, while further exploration is needed for “when” and “why”. This study focuses on the impact of glutamine metabolic reprogramming on tumor immune evasion, especially its effects on macrophage polarization.

### 2.2 Metabolic reprogramming reshapes TAM

In solid tumors, TAM is abundant, with distinct subpopulations exerting diverse effects on tumor initiation and progression. During the early stages of tumorigenesis, TAM predominantly exhibits pro-inflammatory functions that suppress tumor development. As the tumor progresses, macrophages in the TME undergo “education” by Th2 cells, acquiring an M2 phenotype (16). These M2-polarized TAMs modulate tumor growth through multiple mechanisms, including immunosuppression and immune evasion via TGF-i and IL-10-mediated pathways, VEGF secretion to promote angiogenesis (contributing to chemoresistance), and tumor regrowth following radiotherapy (17). The conventional dichotomous classification of TAM into pro-inflammatory M1 or immunosuppressive M2 phenotypes oversimplifies their heterogeneity across tumor types and developmental stages. Inflammatory macrophages adopt the M1 phenotype upon lipopolysaccharide (LPS) stimulation, whereas anti-inflammatory macrophages polarize toward the M2 phenotype via IL-4 or IL-13 induction. The M2 subset further differentiates into: (1) M2a (induced by IL-4/IL-13), (2) M2b (activated by immune complexes and TLR ligands, also termed regulatory macrophages), which participate in immune and inflammatory responses by secreting both pro- and anti-inflammatory cytokines, and (3) M2c (activated by glucocorticoids or IL-10), primarily exerting anti-inflammatory effects. Additionally, M2d macrophages, triggered by TLR ligands and A2 adenosine receptor agonists, play pivotal roles in tumor progression, angiogenesis, and metastasis (17).Despite the established significance of TAM in tumor biology, the mechanistic understanding of how TAM metabolic reprogramming influences tumor growth and immune responses remains incomplete. Consequently, elucidating the metabolic signatures within TME is critical for developing improved therapeutic strategies.

The metabolic network underlying the induction *in vitro* of macrophage polarization encompasses multiple metabolic pathways. These include glycolysis, the TCA cycle (TCA), fatty acid oxidation (FAO), as well as amino acid metabolism and synthesis (18). A nutritional competition exists between cancer cells and TAM, especially in the context of glucose and glutamine metabolism. This competition is tightly regulated by the mTORC1 signaling pathway in TAM (18). Specifically, glutamine is preferentially allocated to cancer cells, which inhibits the phagocytic activity of TAM. Current research has shown that cancer cells overexpress glutamine fructose-6-phosphate aminotransferase (GFAT). This enzyme consumes glutamine during the synthesis of UDP-GlcNAc. As a result, TAM experiences a glutamine deficiency, and its phagocytic activity is attenuated through the inhibition of mitochondrial fission (19). Adequate glutamine levels are essential for mitochondrial fission and the phagocytosis of cancer cells by TAM. It is possible that glutamine promotes the post-translational modification of mitochondrial membrane proteins in TAM by regulating the level of UDP-GlcNAc (20) (**Figure 1**). This implies that glutamine metabolism may play a role in the clearance of cancer cells by TAM.

However, glutamine synthetase (GS) catalyzes the conversion of glutamate to glutamine. The deletion of the GS gene can prevent the synthesis of glutamine and induce the expression of M1-like gene characteristics, thereby inhibiting the function of this enzyme in TAM. This process can enhance glycolysis, reduce tumor endothelial cell branching, and vascular permeability (21). The accumulation of glutamate caused by GS inhibition may be redirected to the TCA cycle and succinate through the γ-aminobutyric acid pathway (18). Researchers speculate that the uptake of extracellular glutamine is necessary to inhibit certain functions of TAM, while the synthesis of glutamine using intracellular metabolites may be detrimental to the anti-tumor function of TAM. Evidently, the uptake of exogenous glutamine and the synthesis of endogenous glutamine have different effects on the fate of TAM.

## 3 Mechanism of glutamine metabolism remodeling TAM

Glutamine metabolic reprogramming influences macrophage phenotype polarization, functional status, and immune regulatory capacity through a multidimensional regulatory network. The following sections will discuss the precise regulatory mechanisms of glutamine metabolism on macrophage function from three perspectives: epigenetic modification, signal transduction, and metabolic checkpoint regulation.

### 3.1 Epigenetic regulation: metabolites as epigenetic switches

#### 3.1.1 Epigenetic regulation of α-ketoglutarate

**Figure 2:**
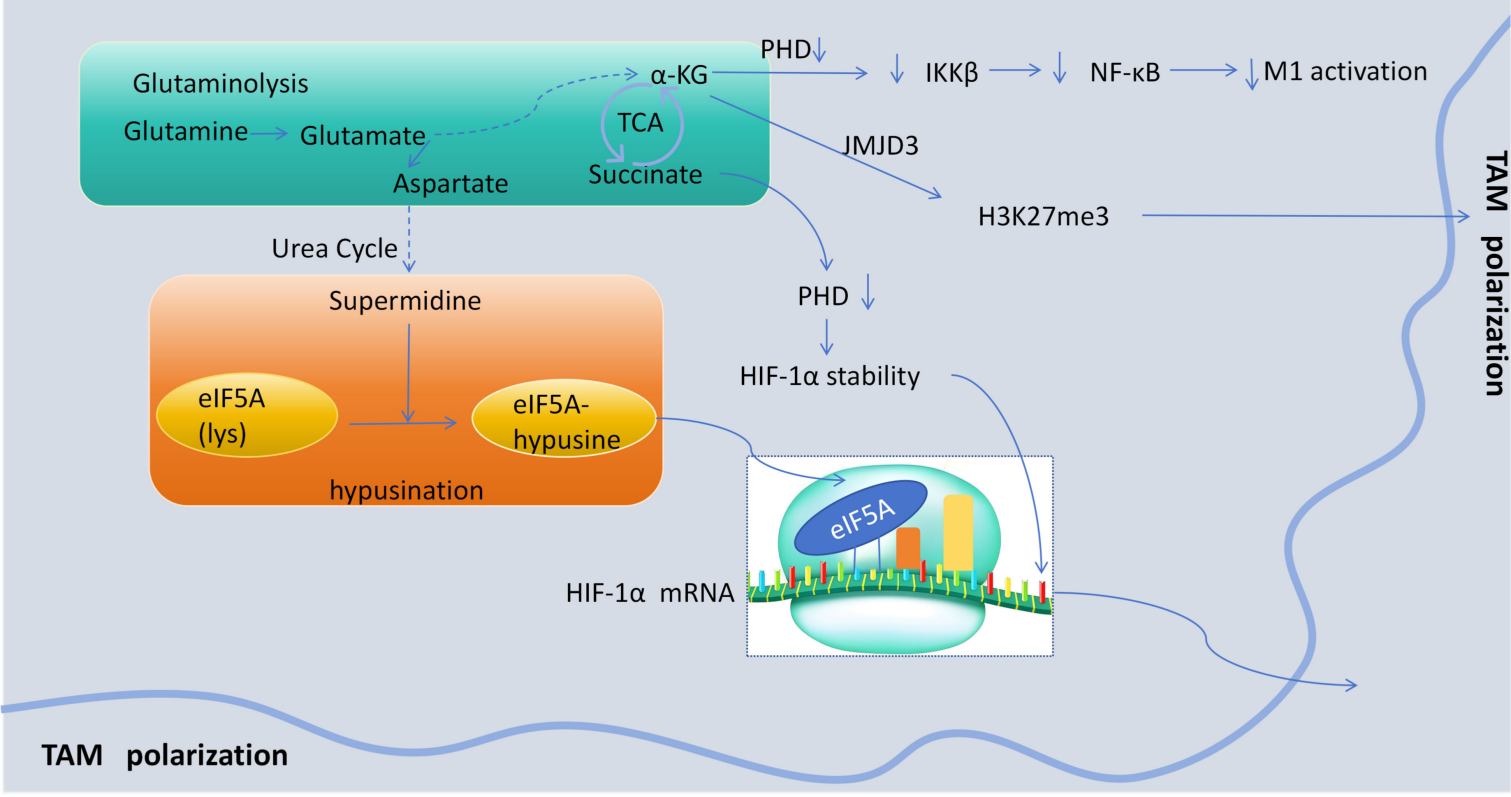
Key processes of glutamine metabolites remodeling TAM polarization.α-Ketoglutarate (α-KG), a metabolite of glutamine metabolism, exerts a pivotal role in macrophage polarization. Moreover, succinate facilitates macrophage remodeling through its impact on the HIF-1α pathway. prolyl hydroxylase (PHD),eukaryotic translation initiation factor 5A (eIF5A).

Glutamine-derived α-ketoglutarate (α-KG) plays a pivotal role in driving macrophage polarization toward an anti-inflammatory, immunosuppressive phenotype (**Figure 2**). α-KG facilitates the transcriptional activation and expression of key immunosuppressive genes, including IL-10, TGFβ, Arg1, Ym1, and Mrc1, through JMJD3-dependent demethylation of H3K27me3 at M2 gene promoter regions (6). Furthermore, by inhibiting prolyl hydroxylase (PHD) activity, α-KG substantially attenuates IKKβ activation (a critical mediator of NF-κB pathway activation)thereby suppressing M1 macrophage functionality (6). Additionally, α-KG stabilizes hypoxia-inducible factor-1α (HIF-1α) through PHD inhibition, modulating TAM activity, particularly in hypoxic regions of the TME (6, 22). In prostate cancer models, JHU083(a glutamine antagonist prodrug)reduces α-KG levels via GLS inhibition, leading to diminished HIF-1α stability and consequent upregulation of proinflammatory signaling in TAM, ultimately promoting their M1 polarization (23).

#### 3.1.2 Glycosylation regulation in the synthesis pathway of hexosamine biosynthetic pathway

In line with the well-established HBP, UDP-GlcNAc is synthesized from glucose, acetyl-CoA, glutamine, and UTP. UDP-GlcNAc then used by OGT to modify proteins with O-GlcNAc. Therefore, UDP-GlcNAc stands as a crucial intermediate that effectively bridges signal transduction and metabolic pathways. Specifically, the inhibition of O-GlcNAc glycosylation modification of transcription factors and signaling proteins leads to a notable reduction in the immunosuppressive function of M2-type macrophages. Through metabolic tracing experiments, it has been conclusively demonstrated that glutamine serves as the primary nitrogen source for UDP-GlcNAc. Consequently, the reprogramming of glutamine metabolism exerts regulatory effects on N-glycosylation modification via UDP-GlcNAc. This, in turn, significantly impacts the polarization process of M2-type macrophages (24, 25) (**Figure 1**).

### 3.2 Signal pathway regulation: cross activation of metabolic-signal networks

#### 3.2.1 The HIF-1α pathway

Succinate, an intermediate metabolite in the TCA cycle, plays a pivotal role in stabilizing HIF-1α in pro-inflammatory macrophages by suppressing PHD activity following LPS stimulation, thereby modulating macrophage polarization (26). In TAM, glutamine-derived aspartate facilitates supermidine production via the polyamine synthesis pathway, which subsequently enhances HIF-1α translational efficiency through eIF5A hypusination. This augmented HIF-1α translation drives TAM toward increased glycolysis, conferring metabolic characteristics distinct from M2 macrophages and ultimately promoting tumor progression (27). These findings demonstrate that succinate-mediated HIF-1α stabilization and glutamine-derived aspartate-enhanced HIF-1α translation collectively regulate macrophage functionality (**Figure 2**). Furthermore, within the TME, tumor cell-derived succinate promotes TAMs polarization and cancer metastasis via the succinate receptor (SUCNR1). SUCNR1 activation triggers the PI3K signaling pathway, consequently amplifying the HIF-1α/VEGF axis in TAM (28).

#### 3.2.2 mTORC1 pathway

Previous research findings have demonstrated that amino acids transmit signals to mTORC1 within lysosomes by activating Rag GTPases. Nevertheless, the newly identified cytoplasmic amino acid sensor suggests that glutamine activates the mTORC1 pathway via a Rag GTPase - independent mechanism that necessitates ADP-ribosyltransferase 1 (ARF1) (29, 30) (**Figure 1**).

It is well - established that cancer cells exhibit the highest consumption of glutamine, whereas immune cells consume relatively less glutamine. In tumor cells, mTORC1 functions as a crucial downstream effector in regulating the metabolic reprogramming of TAM. Evidences from studies have indicated that mTORC1 inhibits autophagy by activating the ULK complex. On the other hand, mTORC1 is involved in responding to energy metabolism and orchestrates the growth factor signal cascade through AMPK proteins and the TSC1 - TSC2 complex (29). Additionally, mTORC1 inhibits autophagy by sequestering the transcription factor TFEB in the cytoplasm (31). These findings underscore the significance of inhibiting the mTORC1 signaling pathway.

### 3.3 Regulation of metabolic checkpoints: the metabolic basis of immunosuppression

#### 3.3.1 Metabolically dependent expression of PD-L1

The glutamine uptake inhibitor V - 9302 has been shown to induce tumor cells to express programmed cell death - ligand 1 (PD-L1). Moreover, when combined with PD-L1 inhibitors, it can synergistically enhance the immune escape capacity of tumor cells in mouse models (32). The underlying mechanism suggests that under glutamine-restricted conditions, cells reduce GSH levels, thereby decreasing the activity of SERCA (sarco/endoplasmic reticulum calcium - ATPase).This triggers the calcium/NF-κB signaling cascade that in turn upregulates the expression of PD-L1 (32). Another study revealed that glutamine deprivation can upregulate the expression of PD-L1 in both renal and bladder cancer cell lines, which occurs through the activation of EGFR/ERK/C-Jun signaling pathway (33, 34).Consequently, glutamine metabolism exerts an impact on the TME by modulating PD-L1 expression. Research on the role of novel immune checkpoints (such as CTLA4,LAG3 and TIGIT)in glutamine metabolism remodeling of TAM is very limited, which might be one of the directions to explore in the future.

#### 3.3.2 Glutamine regulation of the metabolic checkpoints of spermidine

Arginine is a semi-essential amino acid. Under certain physiological and pathological conditions, dietary and renal-derived arginine cannot meet cellular demands, necessitating endogenous synthesis (35). Glutamine metabolism provides nitrogen sources, regulating macrophage immune function by influencing arginine pool distribution. Arginine cooperates with RNA-binding motif protein 39 (RBM39) to control metabolic gene expression. RBM39-mediated upregulation of asparagine synthesis enhances arginine uptake, forming a positive feedback loop that sustains elevated arginine levels and oncogenic metabolism (36). Polyamines derived from arginine metabolism promote TAM polarization toward tumor-promoting phenotypes through thymine DNA glycosylase-mediated DNA demethylation (37). Collectively, these mechanisms establish arginine as a secondary messenger-like molecule that reprograms metabolic regulation of immune cells to facilitate tumor growth.

Glutamine metabolic reprogramming in tumors represents an active regulatory process governed by diverse intra- and extracellular factors, rather than a passive adaptation to proliferative states. These discoveries not only elucidate the metabolic basis of immunosuppression in the TME, but also identify precise therapeutic targets for metabolic-immune interactions. Future research should focus on delineating the spatiotemporal dynamics of metabolic reprogramming, uncovering the molecular basis of metabolic heterogeneity through single-cell technologies, and developing cell-type-specific metabolic modulators to pioneer novel directions in cancer immunotherapy.

## 4 Translational medical strategies aimed at glutamine metabolism

At present, drugs targeting glutamine metabolism are primarily categorized into three groups: glutamine antimetabolites, glutaminase inhibitors, and glutamine uptake inhibitors (**Table 1**). Glutamine antimetabolites encompass DON, JHU083, and DRP - 104. DON (6-Diazo-5-oxo-L-norleucine), as the first-generation glutamine antimetabolite, irreversibly inhibits the active site of glutaminease. It also non -specifically binds to other glutamine-dependent enzymes, demonstrating notable anti-tumor effects. Nevertheless, its systemic toxicity restricts its clinical application (38, 39). JHU083, a prodrug form of DON, selectively activates DON within the TME. This mechanism not only reduces its systemic toxicity but also enhances the anti -tumor immune response (40). Mechanistically, the glutamine antagonistic effect mediated by JHU083 in the TME induces the secretion of tumor necrosis factor (TNF) and pro-inflammatory factors and activates mTORC1 signaling pathway, thereby remodeling TAM. The TAM reprogrammed by JHU083 exhibit augmented tumor cell phagocytic capacity and attenuated angiogenic ability. Additionally, JHU083 can facilitate CD8+ T cells to present a stem cell-like phenotype and reduce the number of regulatory T cells (23). Moreover, blocking glutamine metabolism also inhibits the expression of indoleamine 2,3-dioxygenase (IDO) in tumors and myeloid - derived cells, leading to a substantial decline in kynurenine levels, thus further intensifying the anti-tumor immune response (41). DRP-104, another prodrug form of DON, can comprehensively disrupt glutamine metabolism while evading the toxicity of DON. This results in a tumor metabolic crisis to achieve antitumor effects. The antitumor effects of DRP-104 have been observed in both athymic mice and NSG mice, indicating that the adaptive immune system is not the primary driving force of the antitumor response (42). Based on the preclinical research findings of DRP - 104, it has advanced to the clinical trial stage (NCT04471415).

**Table 1:** Mechanistic role and clinical application of various drugs targeting glutamine metabolism.

Classification	Drug	Role	Clinical application	References
Glutamine antimetabolites	DON(6 - Diazo - 5 - oxo - L - norleucine)	First-generation glutamine antimetabolite function by covalently and irreversibly inhibiting the active site of glutaminase, yet they also exhibit non-specific binding to various other enzymes dependent on glutamine	It has high systemic toxicity, which limits its clinical application	(38, 39)
	JHU083	The prodrug form of DON selectively activates and converts into DON within TME, reprogramming TAM by secreting pro-inflammatory factors and activating the mTORC1 signaling pathway, thereby enhancing anti-tumor immunity	Prostate cancer Bladder cancer	(23, 40, 41)
	DRP-104	The prodrug form of DON can comprehensively impair Gln metabolism, thereby avoiding DON toxicity while preserving its covalent and irreversible properties	Pancreatic cancer NCT04471415	(42)
Glutaminase inhibitors	BPTES(Bis-2-(5-phenylacetamido-1, 3, 4- thiadiazol- 2- yl) ethylsulphide)	GLS allosteric inhibitor, maintaining GLS in an inactive tetrameric state	Primarily used in animal experiments, requiring drug optimization	(43)
	Compound 968	GLS2 inhibitors, which block the GLS2-mediated compensatory metabolic pathway	Breast cancer	(43)
	CB-839	BPTES derivatives with oral bioavailability	Renal cell carcinoma(NCT03428217)	(44–46)
			Triple - negative breast cancer KRAS wild - type metastatic colorectal cancer Non -small cell lung cancer(NCT04250545)	
Glutamine uptake inhibitors	GPNA(L-γ-glutamyl- p-nitroaniline)	The first generation of inhibitors targeting SLC1A5 with low efficacy	Inefficient, limiting clinical application	(47)
	V-9302	Second-generation GPNA derivatives, with efficacy enhanced by 100-fold	Breast cancer	(47, 48)

BPTES, compound 968, and CB-839 are currently the principal GLS inhibitors. Both BPTES and compound 968 are allosteric inhibitors that do not engage in competition with the active site of glutamine. BPTES maintains GLS in an inactive tetrameric state. Compound 968, on the other hand, functions as a GLS2 inhibitor. It can obstruct the salvage metabolic pathway mediated by GLS2 and demonstrates a more potent inhibitory effect on BPTES-resistant cells proliferation and tumorigenesis (43).The co-crystal structure of BPTES and GLS has facilitated drug optimization efforts. As a result, a potent and orally bioavailable derivative of BPTES, namely CB -839, has been developed (44). Due to the absence of biomarkers that could indicate the impact of glutamine metabolism, CB-839 did not enhance the efficacy of cabozantinib in the treatment of resistant metastatic renal cell carcinoma (NCT03428217) (45).However, it exhibited anti-proliferative activity in triple-negative breast cancer cells, but no anti-proliferative effect was observed in estrogen-positive cells (46). Additionally, it was determined that CB-839 is effective whether administered alone or in combination with paclitaxel (46).In patients with KRAS wild-type metastatic colorectal cancer, the combination of CB-839 and panitumumab has shown a favorable initial response (NCT03263429) (9).Multiple studies have revealed a synergistic effect between CB - 839 and mTOR inhibitors. Currently, an ongoing phase I clinical trial (NCT04250545) is exploring the use of the mTOR inhibitor MLN0128 in combination with CB-839 for the treatment of non-small cell lung cancer (44).

The glutamine transporter SLC1A5 plays a pivotal role in glutamine uptake by tumor cells and is frequently upregulated in various cancers. Under conditions of SLC1A5 overexpression, glutamine-dependent tumor cells exhibit poor prognosis, aggressive biological behaviors, and unlimited proliferation (43). Glutamine uptake inhibitors targeting SLC1A5 include the first-generation L-γ-glutamyl-p-nitroaniline (GPNA), which demonstrates limited efficacy, and the second-generation GPNA derivative V-9302, which enhances glutamine uptake inhibition efficacy by approximately 100-fold. In triple-negative breast cancer (TNBC) mouse models, V-9302 suppresses tumor growth by blocking glutamine uptake in TNBC cells without affecting T cells. Mechanistic studies reveal that V-9302 sustains CD8+ T cell glutamine uptake capacity by promoting compensatory upregulation of the glutamine transporter ATB0,+/Slc6a14 in CD8+ T cells, thereby supporting *de novo* glutathione synthesis and improving T cell redox balance (47). Concurrently, in human breast cancer cell lines, V-9302 induces autophagy by downregulating B7H3 (an immune checkpoint ligand that promotes immune evasion) and enhances antitumor effects by modulating ROS accumulation. Combination therapy with V-9302 and anti-PD-1 monoclonal antibodies significantly improves antitumor efficacy in breast cancer mouse models (48).

In this context, which glutamine blockade strategy proves most beneficial, and which approach synergizes optimally with immune checkpoint inhibitors? To date, clinical trials of glutamine metabolism inhibitors have yielded limited progress. Selective modulation of immune cell metabolism within the TME remains an ongoing challenge. To mitigate systemic adverse effects, numerous targeted drug delivery systems have emerged. Cell-derived microparticles (MPs), extracellular vesicles measuring 100–1000 nm in diameter, exhibit inherent macrophage-targeting capabilities and represent promising carriers for macrophage-specific delivery. Wei et al. developed mannose-modified macrophage-derived microparticles loading metformin(Met@Man-MP), which remodel the TME by polarizing TAM, enhancing CD8+ T cell recruitment to tumor tissues, and reducing myeloid-derived suppressor cell and regulatory T cell infiltration (49). Similarly, Kang et al. engineered hollow iron oxide nanoparticles loaded with the small-molecule PD-1/PD-L1 inhibitor BMS-202 and NaHCO3, which reprogram M2 TAM to M1 TAM, block the PD-1/PD-L1 pathway, and subsequently activate T cells to secrete TNF-α and IFN-γ, thereby eliminating tumor cells (50). An ideal drug encapsulation system could not only precisely deliver glutamine metabolism inhibitors to circumvent systemic toxicity but also modulate TAM and even ameliorate the acidic TME, offering a promising avenue to enhance therapeutic outcomes.

Chimeric antigen receptor T cell (CAR-T) therapy has achieved remarkable success in treating hematologic malignancies such as lymphoma and acute lymphoblastic leukemia, yet its broader application in solid tumors faces significant challenges. Chimeric antigen receptor macrophages (CAR-Ms) may emerge as a breakthrough to overcome CAR-T limitations in solid tumors, leveraging their enhanced tumor infiltration capacity (NCT04660929) and potential metabolic advantages (51, 52). Wang et al. demonstrated that ACOD1 gene knockout in CAR-Ms reduces itaconate levels, preventing NRF2 nuclear translocation and subsequent anti-inflammatory programming via KEAP1, thereby polarizing macrophages toward a proinflammatory state characterized by elevated ROS production, enhanced phagocytosis, and superior tumor suppression (52). Advances in understanding TAM metabolism present new opportunities to explore macrophages metabolic reprogramming for improving macrophage-based adoptive cell therapies (18).

Tissue-resident macrophages exhibit high adaptability, integrating diverse TME signals to drive polarization toward immunosuppressive, pro-tumorigenic, and pro-metastatic functional states (18). Studies indicate that reprogrammed mammary adipose tissue macrophages accumulate in breast adipose tissue, contributing to TNBC tumorigenesis (53). This complexity underscores the challenges in deciphering population-specific macrophage heterogeneity in specific niches. Based on single-cell sequencing technology, we have gained deeper insights into the metabolic diversity of TAM subtypes and their impact on disease pathophysiology. Wei et al. (54) used single-cell sequencing data to identify a TAM subset, APOE+CTSZ+TAM, which was highly prevalent in tumor samples and exhibited immunosuppressive characteristics by upregulation the expression of anti-inflammatory genes. Glutamate-to-glutamine metabolic flux scores and glutamine synthetase (GLUL) expression were significantly higher in APOE+CTSZ+TAM cells compared to other cell types within the TME. However, the aim of analyzing the entirety of cellular metabolites at single-cell resolution is challenging owing to enormous structural diversity, rapid turnover, and low analyte abundances in a limited sample volume. Wang et al. (55) proposed an advanced high spatial resolution metabolomics approach, integrating matrix-assisted laser desorption/ionization mass spectrometry imaging (MALDI-MSI) with isotope tracing, in order to overcome the heterogeneity of cells in the tissue context. Zhang et al. develop a universal dynamic metabolomics system based on stable isotope tracing at the single-cell level. This approach enables comprehensive metabolic analysis of co-cultured tumor cells and macrophages, identifying for the first time diverse polarized subtypes of tumor-associated macrophages based on metabolic signatures (56).Advances in technologies such as untargeted metabolomics at single-cell resolution, spatial transcriptomics, and organoid microfluidic chips are transforming TAM metabolic research from static investigations into dynamic, spatially resolved, and functionally stratified analyses across disease stages, offering deeper insights into tumor biology. Furthermore, artificial intelligence may yield advanced algorithms to predict patient-specific metabolic vulnerabilities and guide personalized therapeutic strategies.

## 5 Conclusions

Glutamine plays a pivotal role in energy metabolism and the synthesis of biological macromolecules, underscoring its significance in tumor progression and immune responses. Tumor cells exhibit a pronounced dependence on glutamine. Selectively inhibiting the glutamine metabolism of tumor cells holds promise in alleviating metabolic competition within the TME, thereby liberating glutamine for utilization by immune cells. Glutamine metabolism remodels the functions of TAM via epigenetic and metabolic checkpoint interactions, positioning it as a key target for reversing the immunosuppressive microenvironment. In-depth exploration of hitherto unknown metabolic therapeutic targets in TAM and the development of novel drugs capable of selectively targeting and modulating TAM glutamine metabolism are of paramount importance. This approach is essential for eliciting a sustained anti - tumor immune response while minimizing adverse reactions. Notably, building upon the latest research regarding the cross-metabolic regulation of glutamine and other nutrients on the immune effects within the TME, conducting clinical trials based on metabolic typing and facilitating the integration of “metabolism-immunity” combination therapies into the realm of precision medicine will be a crucial area of expansion for future research endeavors.
